# Epidemiology of musculoskeletal symptoms, rheumatologic disorders, and disability in the Zoroastrian population in Yazd, Iran: a WHO-ILAR COPCORD study (stage 1)

**DOI:** 10.1186/s41927-021-00214-2

**Published:** 2021-11-02

**Authors:** Ali Dehghan, Hossein Soleimani Salehabadi, Ahmadreza Jamshidi, Zohre Kamali, Mojgan Mali, Seyedeh Tahereh Faezi, Azarakhsh Baghdadi, Sogol Alesaeidi, Fatemeh Sahraei, Niloufar Azizi, Sanaz Zand, Sara Sadat Yasini, Maryam Namazi, Atefe Daya, Ryan Nazemian, Farimah Shamsi, Mohammad Nejadhosseinian, Fereydoun Davatchi

**Affiliations:** 1 Department of Internal Medicine, Shahid Sadoughi Hospital, Shahid Sadoughi University of Medical Sciences, Yazd, Iran; 2 Rheumatology Research Center, Tehran University of Medical Sciences, North Amirabad Street, Tehran, 1411713137 Iran; 3 Diabetic Care Center, Shahid Sadoughi University, Yazd, Iran; 4 Ziayee Hospital, Shahid Sadoughi University, Ardakan, Yazd, Iran; 5 Shahid Sadoughi Hospital, Shahid Sadoughi University of Medical Sciences, Yazd, Iran; 6 Feiz Hospital, Isfahan, Iran; 7 Shahid Sadoughi University of Medical Sciences, Yazd, Iran; 8 Clinical Translational Science PhD Program, Case Western Reserve University, Cleveland, OH USA

**Keywords:** COPCORD, Community-based epidemiology, Rheumatic diseases, Musculoskeletal disorders, Disability, Zoroastrians

## Abstract

**Background:**

The purpose of this study was to determine the prevalence of musculoskeletal complaints, rheumatologic diseases, and disability among the Zoroastrian population in Iran.

**Methods:**

The city of Yazd, in central Iran was selected for this study, with the highest population of Zoroastrians in Iran. Subjects were selected by cluster sampling of 9 neighborhoods populated with Zoroastrians. Subjects ≥15 years old were interviewed by trained interviewers in their houses. The validated Farsi translation of Community Oriented Program for the Control of Rheumatic Disease (COPCORD) Core Questionnaire (CCQ) was used for this study. Subjects with musculoskeletal complaints (pain, stiffness and/or swelling) were examined by a rheumatologist. Laboratory tests and radiographic exams were performed when deemed necessary.

**Results:**

Two-thousand subjects were interviewed during a 12-month period, of which 956 were male, and 1044 were female. The mean age was 41.1 ± 18.3 years (95%CI: 40.3–41.9). 36.9% of the subjects had university-level education. In the 7 days prior to the interview, 27.6% of the subjects had musculoskeletal complaints, with the knee, dorsolumbar spine, and shoulder being the most common sites of complaints. The most common rheumatologic diagnoses were osteoarthritis (21.5%) and low back pain (10.3%). Rheumatoid arthritis was diagnosed in 1.2% of the subjects.

**Conclusions:**

The epidemiology of musculoskeletal complaints and rheumatologic disorders was inconsistent with previous COPCORD studies in Iran, with a lower prevalence of musculoskeletal complaints in general, lower rates of Behçet and lupus, and a higher prevalence of rheumatoid arthritis. The findings of this study can be for development of better prevention, screening, and treatment programs for the vulnerable population of Zoroastrians in Iran.

## Background

The Community Oriented Program for Control of Rheumatic Diseases (COPCORD) was established in 1981 by WHO (World Health Organization) and ILAR (International League of Associations of Rheumatology), focusing on pain and disability caused by rheumatologic disorders in the developing countries. This initiative was launched with the aims of recognition, prevention, and control of rheumatologic disorders in communities with limited infrastructure and financial resources. To date, 21 countries have undertaken stage 1 of the program, which sought to evaluate at least 1500 people over 15 years of age. While being a non-governmental project relying on local funds and resources, COPCORD has proven to be an outstanding endeavor to improve our understanding of the burden of musculoskeletal disorders in communities with the least access to healthcare. More information about the program’s agenda is available on the COPCORD website (www.copcord.org).

Iran is situated in the crossroads of the East and West and is in the middle of the ancient silk route. Due to its unique geographic, demographic, and political situation in the world, as well as good healthcare infrastructure, Iran was an interesting addition to the COPCORD project. To date, multiple COPCORD studies have been performed in Iran as part of stage 1 of the project. A pilot study [1] was followed by an urban study in Tehran [2]. Tehran, the capital city of Iran, was selected as the first urban center for the study, mainly because it represented all ethnic groups in the country and the availability of resources [2]. With 10,291 subjects, it was one of the most successful COPCORD studies to date and was followed by a rural study in Tuyserkan [3]. Zahedan, a city in the less developed southeast of Iran, was the subject of the second urban study [4]. Sanandaj, situated in northwestern Iran, with a mainly Kurd population, was the next COPCORD urban field [5]. The fourth study was performed in Yazd, and the combined data from all four urban COPCORD sites have been analyzed [6].

As a protected minority group, the Zoroastrian community has inhabited central Iran, particularly the Yazd province, for centuries. While efforts have been made to promote inclusion and diversity, discrimination still exists against protected communities. The prevalence of mental illnesses, including depression and anxiety, has a higher incidence among Zoroastrian communities [7]. The purpose of this study was to report the results of the fourth urban COPCORD study, conducted among the Zoroastrian population in Yazd, Iran, to determine the prevalence of musculoskeletal complaints, rheumatologic diseases, and disability among the Zoroastrian population in Iran.

## Methods

Yazd, the capital city of the Yazd province, was selected for this study. Yazd, with a population of 529,673, was selected due to its unique ethnic distribution. Iran has a heterogeneous ethnic distribution overall, with 75.4% of the population being Caucasians and around 22% Turks. Semites constitute the third-largest population, with 2.6% of the population, and include Arabs, Jews, and Assyrians. Zoroastrians are another Caucasian minority, mainly concentrated in Yazd. While most of the population in Iran are of mixed descent, the Zoroastrian population of Yazd has maintained a semi-closed community with a very low rate of intermarriage. The Zoroastrians have not been the subject of a community-based study before, with a knowledge void regarding the epidemiology of diseases, particularly common musculoskeletal symptoms and rheumatologic disorders.

### Sampling plan

The population of Zoroastrians in Iran stood at 25,271 according to the latest census results, with the vast majority residing in the Yazd province [8]. Nine neighborhoods with a high Zoroastrian population were chosen, and subjects were recruited by cluster sampling in those neighborhoods.

### Questionnaire

The Farsi translation of the COPCORD Core Questionnaire (CCQ) was used to screen subjects for musculoskeletal complaints [9]. This translation has been validated and has been shown to be reliable and reproducible [1]. The following sections of the CCQ were administered to all individuals as described previously: sections A (background information), B (work history), C1 (pain, tenderness, or stiffness in the last week), D (functional disability), G (evaluation), and H (extra-articular symptoms of rheumatic diseases (aphthous ulcers, blurred vision, etc.), including Behçet’s disease) [1, 2, 5]. The detailed methodology of subject selection and administration of the test have been reported previously [1].

### Data collection

Two physicians (general practitioners) who knew the local community were trained on the details of COPCORD methodology at the Rheumatology Research Center of Tehran University of Medical Sciences. A training workshop was held for 12 interviewers in Yazd by the practitioners and a member of the COPCORD team from the Rheumatology Research Center. Also, Bachelor students from Yazd University of Medical Sciences were recruited to supervise the data collection team and monitor the process, fill the special evaluation checklist, and manage the groups.

### Pilot study

A Pilot study was performed to evaluate the possibility and limitations of the COPCORD study. Two members from the RRC in Tehran who were familiar with the study model travelled to Yazd to overlook the pilot study. Fifty subjects were randomly selected. The Pilot study was carried out on the weekend (Friday in Iran). The selected individuals were informed about the goals of the study, and were then interviewed by the team. Subjects who had musculoskeletal complaints or a history of oral ulcer were referred to the clinic for further evaluation. Two rheumatologists were present in the clinic and evaluated the patients, including a detailed history and physical examination, and laboratory and imaging studies if needed.

### Data collection

The selected cluster was visited by the team consisting of the project manager, a rheumatologist, interviewers, and a lab technician, 2 days each week. Households were selected randomly within each cluster based on postal codes, and a minimum of 250 subjects were interviewed in each cluster to ensure a random distribution. The identified households were visited, and a thorough explanation about the study was given. Then, a form was completed for each family, asking about the information of household members 15 years of age or older. For absent individuals, the team went back to the same household for two consecutive days to collect data if possible. Filled questionnaires were then forwarded to the team head, to be checked for quality of data collection and refer individuals who needed a physical examination to the rheumatologist. Blood samples were taken during the same visit if necessary. Similar to previous COPCORD studies, rheumatologic diseases and syndromes were diagnosed based on their accepted criteria (SLE, Behçet’s disease, RA, etc.), while mechanical disorders (e.g., osteoarthritis, rotator cuff disorders) were assigned based on the visiting rheumatologist’s diagnosis [6].

### Quality control

All interviewers underwent regular quality control checks from the project manager. Furthermore, all of the forms and examination sheets were checked by the team head in the field. CCQs were rechecked later and were evaluated for any missing data or errors.

### Data analysis

Descriptive data were analyzed by survey data analysis methods with regard to age, sex, and weight of the cluster. Statistical analysis was performed with IBM SPSS Statistics for Windows, version 23.0 (IBM, Armonk, NY) and STATA (StataCorp, College Station, TX, USA). The prevalence of musculoskeletal disorders was adjusted to age-sex distribution of the study population from the 2011 Census [10]. Prevalence rates were presented as percentages (95% confidence interval [CI]).

### Power

Previous COPCORD studies performed in Iran have shown a 30–50% prevalence of musculoskeletal complaints, 15–25% for degenerative joint diseases, and approximately 1% for rheumatologic disorders [6, 11, 12]. Therefore, with a 5% type I error and an absolute error of 5%, this study would need > 138 individuals for a disease with 10% prevalence (e.g., musculoskeletal complaints), and > 1382 individuals for 1% prevalence (e.g., rheumatologic diagnoses). Therefore, a minimum sample size of 1500 was set for this study.

## Results

**Fig. 1:**
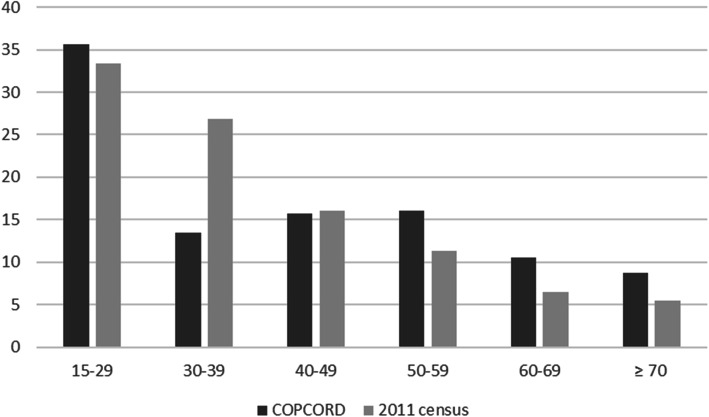
Age distribution in this study compared to the 2011 census. Horizontal axis denotes the age group in years, and the vertical axis is percent

The data collection phase was completed in 12 months. At the end of the study, 2000 eligible subjects were interviewed. Among them, 645 (374 female, 271 male) needed a physical examination by a rheumatologist. The male to female ratio was 0.91:1, with 956 (47.8%) male and 1044 (52.2%) female subjects. The 2011 Yazd census showed a 1.06:1 male (51.4%) to female (48.6%) ratio. All subjects in this study were Caucasians of Zoroastrian ancestry. The age distribution in comparison to the 2011 census is illustrated in Fig. 1, with gender details in Table 1.

**Table 1:** Age distribution categorized by gender

Age (years)	Male				Female			
	N	%	95% CI	Census %	N	%	95% CI	Census %
15–29	369	38.6	35.4–41.8	33.2	342	32.8	29.9–35.7	33.7
30–39	128	13.4	11.2–15.5	27.1	141	13.5	11.5–15.5	26.8
40–49	137	14.3	12.1–16.4	16.5	177	17	14.6–19.1	15.9
50–59	138	14.4	12–16.7	11.6	184	17.6	15.3–19.9	11.1
60–69	101	10.6	8.6–12.6	6.4	108	10.3	8.5–12.2	6.6
≥ 70	83	8.7	6.9–10.6	5.2	92	8.8	7.2–10.6	5.9
Total	956	100			1044	100		

### Education level

1.1% of the subjects were illiterate. 25.7% had some primary school to some high school education, 36.2% had a high school diploma, and 37% of the subjects had a university-level education

### Musculoskeletal complaints

Overall, 27.6% of the subjects had at least one episode of musculoskeletal complaints during the last 7 days of the interview, which included patients with continuous or episodic complaints. Musculoskeletal complaints were more frequent among females (37.1%) compared to males (17.2%). The interviewees were also asked for musculoskeletal complaints in the past (> 7 days ago), of which 19.1% answered positively. Table 2 summarizes the details of musculoskeletal complaints broken down by age groups.

**Table 2:** Musculoskeletal complaints in the subjects of this study, broken down by age, and complaint

Age	Overall complaints		Pain		Swelling		Stiffness	
	%	CI	%	CI	%	CI	%	CI
15–29	9	7–11.1	9	6.9–11	1.3	0.6–2.1	2	1–3.1
30–39	18.2	13.8–23	18.2	13.8–23	3	1.1–5.2	3.7	1.5–5.9
40–49	33.4	28–38.9	32.8	27.7–37.9	7.6	5.1–10.8	11.5	8.3–15.6
50–59	39.4	33.9–45.3	38.2	32.9–43.5	12.7	9–16.5	13.7	9.9–17.4
60–69	48.8	42.1–56.5	48.8	41.6–56	19.1	14.4–24.9	17.2	12–22.5
≥70	59.4	52–66.3	59.4	52–66.9	22.9	16.6–29.7	21.7	16–28
**All ages**	27.6	25.8–29.5	27.3	25–29	8.1	6.7–9.5	8.9	7.8–10.3

*CI* 95% confidence interval

### Joint distribution of musculoskeletal complaints (pain, swelling, and stiffness)

Shoulder 9.3% (95% confidence interval: 7.9–10.6), elbow 4.4% (95% CI: 3.4–5.3), and wrist 5.1% (95% CI: 4.2–6.1), were the most common sites of musculoskeletal complaints. Details and gender-specific data is summarized in Table 3.

**Table 3:** Gender distribution of musculoskeletal complaints based on site of symptoms

Joint	Men	Women	All
Shoulder	4.6 (3.3–6.1)	13.6 (11.5–15.6)	9.3 (7.9–10-6)
Elbow	2.2 (1.4–3.1)	6.3 (4.8–7.9)	4.4 (3.4–5.3)
Wrist	1.8 (1–2.7)	8.2 (6.5–9.9)	5.1 (4.2–6.1)
Hand	2.4 (1.5–3.5)	10.2 (8.3–12.1)	6.5 (5.5–7.6)
Hip	2.6 (1.7–3.8)	8.3 (6.7–10.2)	5.6 (4.6–6.6)
Knee	9.9 (7.9–11.9)	23.2 (20.7–25.8)	16.9 (15.3–18.5)
Ankle	2.8 (1.9–4.1)	7.8 (6.1–9.6)	5.4 (4.4–6.4)
Foot	1.9 (1–2.7)	6.6 (5.2–8.2)	4.4 (3.5–5.3)
Cervical Spine	4 (2.8–5.3)	12.7 (10.7–14.6)	8.5 (7.3–9.8)
Dorsolumbar	7.6 (5.9–9.4)	19.3 (17–21.6)	13.7 (12.2–15.2)
**All sites**	**17.2 (14.6–19.6)**	**37.1 (34–40.3)**	**27.6 (25.8–29.5)**

Data is presented as percent (95% confidence interval)

In patients who had musculoskeletal complaints in the past (> 7 days prior to the interview), knee (17.3%), dorsolumbar spine (15.9%), and shoulder (10.4%) were the most common complaint sites.

### Diagnosed diseases

Degenerative joint disease was diagnosed in 21.5% of the subjects, with knee osteoarthritis being the most common site in both males and females, followed by hip and neck. Among other mechanical disorders, low back pain was the most prevalent (10.3, 95%CI: 9–11.6), followed by sciatica (5.1, 95%CI: 4.2–6.2). The prevalence of tendinitis and bursitis in total was 5.2% (95%CI: 4.3–6.2). Inflammatory rheumatologic diseases diagnosed in the subjects included Rheumatoid Arthritis (1.2, 95%CI: 0.8–1.7), seronegative spondyloarthropathies (0.7, 95%CI: 0.4–1.1), fibromyalgia (0.4, 95%CI: 0.1–0.6), and gout (0.3, 95%CI: 0.1–0.5). Of note, no cases of systemic lupus erythematous and Behçet disease were diagnosed in this study. Table 4 summarizes the diagnosed diseases in this study.

**Table 4:** Diagnosed diseases in this COPCORD study

Diagnosis	Male % (95% CI)	Female % (95% CI)	All % (95% CI)
**Osteoarthritis**			
Total	12.7 (10.8–15)	29.5 (26.9–32.4)	21.5 (19.7–23.3)
Hip	4.1 (2.9–5.3)	9.8 (8.1–11.7)	7.1 (6–8.2)
Knee	8.8 (7.1–10.5)	19.7 (17.4–22.1)	14.5 (12.9–16)
Hand	2.1 (1.2–3)	6 (4.6–7.6)	4.2 (3.3–5)
Neck	2.5 (1.6–3.5)	10 (8.2–11.8)	6.4 (5.4–7.6)
**Other mechanical disorders**			
Chondromalacia Patella	1.5 (0.7–2.4)	1.6 (0.9–2.4)	1.6 (1–2.1)
Low back pain	7.2 (5.6–8.8)	13 (11–15.3)	10.3 (9–11.6)
Carpal Tunnel Syndrome	0.8 (0.3–1.5)	3.8 (2.8–5)	2.4 (1.8–3)
Trigger finger	0.3 (0–0.7)	0.5 (0.1–1)	0.4 (0.2–0.7)
De Quervain Tenosynovitis	0.3 (0–0.7)	0.7 (0.2–1.2)	0.5 (0.3–0.9)
Tennis elbow	0.4 (0.1–0.8)	1.6 (0.9–2.6)	1.1 (0.7–1.6)
Golf Elbow	0	0.4 (0.1–0.8)	0.2 (0.1–0.4)
Shoulder rotator cuff pathology	1 (0.4–1.8)	2.9 (2–3.8)	2 (1.5–2.7)
Sciatica	3.1 (2.1–4.3)	7 (5.7–8.6)	5.1 (4.2–6.2)
Cervical radiculopathy	0.2 (0–0.5)	0.1 (0–0.3)	0.2 (0–0.4)
All Periarthritis	3.1 (2.1–4.2)	7.1 (5.1–8.6)	5.2 (4.3–6.2)
**Inflammatory/pain disorders**			
Fibromyalgia	0.1 (0–0.3)	0.6 (0.1–1.1)	0.4 (0.1–0.6)
Gout	0.2 (0–0.5)	0.3 (0–0.7)	0.3 (0.1–0.5)
Spondyloarthropathy	0.9 (0.4–1.6)	0.4 (0.1–0.8)	0.7 (0.4–1.1)
Rheumatoid Arthritis	0.2 (0–0.5)	2.1 (1.2–3.1)	1.2 (0.8–1.7)
Systemic Lupus erythematous	0	0	0
Behçet disease	0	0	0

*CI* 95% Confidence interval

### Disability

At the time of the interview, 254 subjects (12.7, 95%CI: 11.5–14.3) reported some disability in performing activities of daily living (ADL) caused by musculoskeletal symptoms. Disability was more prevalent in females (18, 95%CI: 15.5–20.2) than males (6.9, 95%CI: 5.4–8.6). Details of ADL broken down by the severity of disability are summarized in Table 5.

**Table 5:** Disability in activities of daily living among subjects in this study

ADL	None		Mild		Moderate		Severe	
	%	CI	%	CI	%	CI	%	CI
Dressing	93.1	91.7–94.7	5.4	4.4–6.5	1.5	1–2.1	–	–
Getting up	87.5	86.3–88.8	9	7.8–10.2	3.3	2.5–4.1	0.2	0.1–0.4
Drinking	98.1	96.5–99.8	1.5	1–2	0.3	0.1–0.5	0.1	0–0.2
Eating	98.2	96.5–99.9	1.3	0.8–1.8	0.4	0.2–0.8	0.1	0–0.2
Walking	89.2	87.7–90.6	7.6	6.5–8.7	3.1	2.4–3.9	0.1	0–0.2
Bathing	14.8	93.5–96.7	3.5	2.6–4.3	1.3	0.8–1.8	0.1	0–0.2
Turkish toilet (squat toilet)	88.1	86.8–89.4	4.5	3.6–5.4	2.3	1.7–2.9	5.1	4.2–6.2
Taking an object on the floor	89	69.6–90.4	7.4	6.3–8.5	2.9	2.2–3.7	0.7	0.3–1
Hanging cloths on a rope	93.9	92.3–95.5	3.8	2.9–4.7	2	1.4–2.6	0.3	0.1–0.5
Going in and out a transport vehicle	88.8	87.6–90.2	8.2	7–9.4	2.9	2.2–3.7	0.1	0–0.2
Cross leg sitting	84.3	83.1–85.5	3.8	2.9–4.6	4.5	3.7–5.5	7.4	6.3–8.6
Praying	95.8	94.2–97.4	3.3	2.5–4	0.8	0.4–1.2	0.1	0–0.2
Opening boxes	96.5	94.9–98.2	2	1.4–2.7	1.1	0.7–1.6	0.4	0.2–0.8

*ADL* activities of daily living, *CI* 95% confidence interval

## Discussion

This study aimed to determine the prevalence of musculoskeletal complaints and rheumatologic disorders among the Zoroastrian population in Yazd, Iran. To the best of our knowledge, this is the first study of its kind in this population. The Zoroastrians are a protected minority in Iran, along with Armenians, Assyrians, and Persian Jews. The large majority of Zoroastrians in Iran live in the Yazd city, and due to generations of relative inbreeding, they have maintained their unique genetic makeup of their Aryan ancestors. In the last decades, low birth rates have affected the growth of this population in Iran. The results of this study contribute to the knowledge of the epidemiology of musculoskeletal symptoms, rheumatologic disorders, and disabilities in this vulnerable population.

The age distribution in this study was slightly different than the 2011 census results, and as such, data were adjusted for age and sex. It should be noted that the mean age of the Zoroastrian population in Iran is higher than the average population and is not due to a sampling error. Interestingly, 36.9% of the interviewees in this study had higher education levels (university-level education). This is higher than the previous COPCORD studies in Tehran (19.9%) [2], Sanandaj (21.5%) [5], and Zahedan (16.9%) [4], and is twice as high as the national average [13].

Musculoskeletal complaints during the 7 days preceding the interview were observed in 27.6% of the subjects, with pain being the most common complaint (27.3%). Among the Iran COPCORD studies, this is the lowest prevalence of musculoskeletal complaints [1–5], and is more in line with Australia [14], Kuwait [15], and Mexico [16] data. Knee, followed by dorsolumbar spine and shoulder, was the most common site of a rheumatologic complaint. This has been a consistent finding in previous Iranian and international COPCORD studies, which highlights the high strains the current living conditions place on the knee (Table 6).

**Table 6:** Previous COPCORD studies, including the studies performed in Iran

	No.	Pain	LBP (%)	OA (%)	Knee OA (%)	STR (%)	FM (%)	RA (%)	SPA (%)	CTD (%)	Gout (%)
Australia [14]	1437	34	22	8.2	15	5.8	–	0.7	0.21	–	1.5
Australia Aboriginal [17]	847	33	12.5	5.5	11.2	7.4	–	0	0.5	0	4
Bangladesh rural [18]	2635	26.9	6.6	–	7.5	2.7	4.4	–	–	–	–
Bangladesh urban slum [18]	1317	24.9	9.9	–	9.2	2.5	3.2	–	–	–	–
Bangladesh urban affluent [18]	1259	27.9	9.2	–	11.5	3.3	3.3	–	–	–	–
Brazil [19]	3038	30.9	–	4.1	–	–	–	0.46	–	–	–
China Shanghai [20]	6584	–	5.6	–	4.1	3.4	–	0.47	0.11	0.06	0.22
China Beijing [21]	4192	40.3	35	–	30	–	–	0.34	0.26	0.01	–
Cuba [22]	3155	43.9	11.6	20.4	–	6.4	0.22	1.2	0.19	–	0.38
Guatemala urban [23]	4000	9.3	–	1.6	–	1	–	0.5	–	–	–
Indonesia Urban [24]	1071	–	23.3	–	–	–	–	0.3	–	–	–
India Bhigwan [25]	4092	18.2	11.4	5.6	3.9	5.5	–	0.5	–	–	0.12
Iran Pilot study [1]	284	34.5	22.2	14.5	26.1	2.4	–	–	–	–	–
Iran Tehran urban [2]	10,291	41.9	15.4	16.6	15.3	4.6	0.7	0.33	0.23	–	0.13
Iran Tuyserkan rural [3]	1565	66.6	23.4	20.5	19.3	2.2	0.06	0.19	1.1	–	–
Iran Zahedan urban [4]	2100	54.1	19	20.7	17	4.8	2.7	1	0.23		0.09
Iran Sanandaj urban [5]	5830	42.8	16.5	19.4	18.8	5.5	0.6	0.51	0.22	0.08	0.12
Kuwait [15]	7670	26.8	–	–	–	–	–	–	–	–	–
Lebanon [26]	3530	32.9	–	4	3	5.8	–	1	0.3	–	0.01
Malaysia [27]	2594	21.1	11.6	–	–	–	–	0.15	0.12	–	–
Mexico [16]	19,213	25.5	–	10.2	–	3.8	–	1.5	0.1	–	0.35
Pakistan [28]	2090	14.8	1.9	3.7	1.8	1.9	2.1	0.55	0.1	0.05	0.14
Philippines urban [29]	3006	–	2.1	4.1	1.4	3.8	0.2	0.17	0.03	–	0.13
Thailand [30]	2463	17.6	4	11.3	5.7	1.5	–	0.12	0.12	0.08	0.16
Vietnam [31]	2119	–	11.2	4.1	18.2	15.4	–	0.28	–	0.09	0.14

*LBP* low-back pain, *OA* osteoarthritis, *STR* soft-tissue rheumatism, *FM* fibromyalgia, *RA* rheumatoid arthritis, *SPA* seronegative spondyloarthropathies, *CTD* connective tissue disease

Along the same lines, osteoarthritis was found to be the most common rheumatologic disease in this study, with a total of 21.5% prevalence, which was similar to previous Iran COPCORD studies. However, international COPCORD studies have a consistently lower prevalence of OA, which might be the result of heterogeneous criteria for the diagnosis of the condition. OA of the knee and hip cause the greatest burden to the population due to pain and stiffness [32]. Knee and hip were the most common sites of OA in this study, similar to previous studies.

Among other mechanical disorders, low back pain was a common diagnosis, and with a 10.3% prevalence, is substantially lower than previous Iran COPCORD studies [1–5], and is more similar to Cuba [22], India [25], Malaysia [27], and Vietnam [31] studies. The Zoroastrians in Iran are highly educated and are less employed in manual labor jobs, which might be the reason for the relatively low rate of low back pain.

Rheumatoid arthritis was the most common inflammatory disease diagnosed in this study. With a prevalence of 1.2%, RA has the highest prevalence in the Iran COPCORD studies, and most international studies. Only Mexico has reported a higher rate (1.5%) [16], and Cuba has also reported a similar prevalence (1.2%) [22]. Interestingly, gout was also more common in this study compared to previous Iranian studies. We found a prevalence of 0.4% for gout, with higher rates only reported in the Australian COPCORD studies [14, 17]. This might be the result of a higher socioeconomic status of the Zoroastrians, with higher consumption of meat and other gout risk factors. Spondyloarthropathies were also common in this study (0.7%) compared to previous Iran and international studies, which might be the result of the genetic makeup of this population, which is different than other Iranian ethnicities.

We did not find a single case of Behçet disease and SLE in this study, which was not the case with the previous COPCORD studies performed in Iran [1–5]. Iran has a high prevalence of Behçet disease [33]. Genetics play a significant role in Behçet disease [34], which again might be the reason for our findings.

The prevalence of disability in performing ADL was 12.7% in this study, with getting up and going in and out of a transport vehicle being the most common causes of mild disability, cross leg sitting and getting up the most common causes of moderate disability, and cross leg sitting, and Turkish toilet (squatting) were the most common causes of severe disability. Of note, the reported disability in this study was lower than previous COPCORD studies in Iran, which was reported at 22.4% in Zahedan [4], and 28.3% in the Sanandaj study [5].

This study has some limitations, including those inherent to the COPCORD methodology. The interview is time-consuming and exhaustive, and the accuracy of the responses may diminish with time. Also, although the CCQ is unique and has been standardized, some differences have been noted in the responses. For example, non-traumatic and traumatic pain have been interchangeably used in some studies, which weakens the reliability of the questionnaire. Additionally, the census data used to adjust data in this study were derived from the Yazd province records, which might be different than that of the Zoroastrian community. Finally, musculoskeletal complaints are subjectively surveyed, and mechanical disorders (e.g., osteoarthritis) is diagnosed by the visiting physician, with no predetermined criteria, which imposes some heterogeneity and bias to the study [35]. The strengths of this study include surveying a high percentage of the community (2000 out of 25,271 people). Also, a high response rate was observed, and a low dropout and refusal rate was recorded.

## Conclusions

In conclusion, the results of this study suggest a distinct epidemiology of musculoskeletal complaints, rheumatologic diseases, and disability in the Zoroastrian population in Iran, with a lower rate of musculoskeletal complaints, osteoarthritis, and Behçet disease, and higher rates of gout and rheumatoid arthritis, compared to other studied populations in Iran. These findings can be used by local and national governments for the development of better prevention, screening, and treatment programs for the vulnerable population of Zoroastrians in Iran. Also, the unique genetic makeup of this population might be the subject of future studies on the genetic predisposition to common rheumatologic disorders.

## Data Availability

The datasets used and/or analyzed during the current study are available from the corresponding author on reasonable request.

## Abbreviations

WHO: World Health Organization
ILAR: International League of Associations of Rheumatology
COPCORD: Community Oriented Program for Control of Rheumatic Diseases
CCQ: COPCORD Core Questionnaire
MSK: Musculoskeletal
ADL: Activities of daily living
OA: Osteoarthritis
RA: Rheumatoid arthritis
SLE: Systemic lupus erythematous
LBP: Low-back pain
STR: Soft-tissue rheumatism
FM: Fibromyalgia
SPA: Spondyloarthropathy
CTD: Connective-tissue disease
